# Effect of Topical Brimonidine 0.15% on Conjunctival Injection after Strabismus Surgery in Children

**DOI:** 10.1155/2021/5574194

**Published:** 2021-05-04

**Authors:** Dong Hyun Kim, Hee Kyung Yang, Sang Beom Han, Jeong-Min Hwang

**Affiliations:** ^1^Department of Ophthalmology, Seoul National University College of Medicine, Seoul National University Bundang Hospital, Seongnam, Republic of Korea; ^2^Department of Ophthalmology, Kangwon National University College of Medicine, Kangwon National University Hospital, Chuncheon, Kangwon, Republic of Korea

## Abstract

**Purpose:**

To investigate the effects of topical brimonidine 0.15% instillation on conjunctival injection after strabismus surgery in children.

**Methods:**

We retrospectively analyzed 63 Korean children who underwent strabismus surgery for intermittent exotropia. Patients received topical brimonidine 0.15% after surgery for up to 4 weeks. Conjunctival injection was objectively assessed using a software that automatically scored the region of interest from the image of the bulbar conjunctiva. Conjunctival injection scores were compared with those of the control group who were not prescribed topical brimonidine.

**Results:**

The mean scores of conjunctival injection after rectus muscle recession and resection were significantly lower in the brimonidine group than the controls at 4 weeks after surgery (*P* = 0.008  and  0.046, respectively). There was no significant difference in intraocular pressure between the two groups. No adverse effects, such as dry mouth, fatigue/drowsiness, headache, sedation, hypotension, or bradycardia, were reported.

**Conclusions:**

Administration of topical brimonidine 0.15% after strabismus surgery is efficacious and safe in reducing postoperative conjunctival injection.

## 1. Introduction

Various complications may occur after strabismus surgery, including local issues such as conjunctival injection, scar formation, and inclusion cysts; however, severe problems such as cellulitis and endophthalmitis are rarely seen [1–3]. Conjunctival injection is by far one of the most common complications after strabismus surgery [2]. Although patients do not expect conjunctival injection to persist for a long time after surgery, it may cause anxiety, cosmetic problems, and disappointment, despite successful ocular alignment [4].

Brimonidine tartrate is a selective *α*2-adrenergic receptor agonist that has been widely used to lower intraocular pressure (IOP) [5, 6]. Alpha-adrenergic agonists bind to *α*-receptors on vascular smooth muscles and induce smooth muscle contraction and vasoconstriction [7]. Alpha-adrenergic agonists have been widely used in the treatment of glaucoma because vasoconstriction limits blood flow to the ciliary muscle and reduces the production of aqueous humor [8]. As brimonidine affects vasoconstriction primarily via the *α*2-adrenergic receptor, it has been reported that the pre- and postoperative use of topical brimonidine can help reduce bleeding-related problems in ophthalmic surgery [9–11]. In addition, brimonidine has relatively lower systemic adverse effects than other vasoconstrictors such as phenylephrine, and the safety of brimonidine has been confirmed in children over 2 years of age [12].

A few studies have assessed the effect of prophylactic topical brimonidine in reducing intraoperative bleeding in strabismus surgery [13–15]. However, to the best of our knowledge, no studies have reported the effect of postoperative use of topical brimonidine in reducing conjunctival injection after surgery. Therefore, we performed this study to evaluate the effect of brimonidine on conjunctival injection after strabismus surgery in children.

## 2. Methods

### 2.1. Patients

A retrospective study was performed on consecutive children between 7 and 12 years of age with exotropia who underwent unilateral lateral rectus recession with medial rectus resection (RR) by the same surgeon (J-M. H.). A limbal conjunctival incision was made in all operations, and all surgical incisions were repaired with a minimal number of sutures with absorbable 8-0 polyglactin sutures (8-0 Polysorb; Covidien, Mansfield, MA, USA). All patients underwent complete ophthalmological examination before surgery. Data were collected on demographics and clinical characteristics, including age, sex, preoperative angle of deviation, dosage of surgery, and cycloplegic refractive errors. Patients were excluded if they had other possible causes of inflammatory diseases of the anterior segment, including conjunctivitis, keratitis, and uveitis, a history of prior strabismus surgery, simultaneous oblique or vertical muscle surgery, simultaneous vertical transposition surgery, adjustable surgery, use of biodegradable collagen matrix implant, ocular disease other than strabismus, systemic disorders such as congenital anomalies, neurological disorders, and connective tissue disease, or if they did not comply with postoperative anterior segment photographs. This study was approved by the Institutional Review Board (IRB) of the Seoul National University Bundang Hospital.

After surgery, all patients received gatifloxacin 0.3% (Gatiflo; Handok, Inc., Chungbuk, Korea), topical fluorometholone 0.1% (Fluvin; Taejoon Pharmaceutical, Seoul, Korea), and topical bromfenac 0.1% (Bronuck; Taejoon Pharmaceutical, Seoul, Korea) for 4 weeks. In addition, patients who were prescribed topical brimonidine tartrate 0.15% (Alphagan P; Allergan, Inc., Irvine, CA, USA) twice a day for 4 weeks were defined as the brimonidine group. The control group was defined as those who did not receive topical brimonidine.

### 2.2. IOP Measurement

IOP was measured using noncontact tonometry (ICT-900, KOWA, Japan) 4 weeks after surgery. IOP measurements were repeated until three measurements differed by ≤1 mm Hg, and the average of these three readings was recorded.

### 2.3. Conjunctival Injection

**Figure 1:**
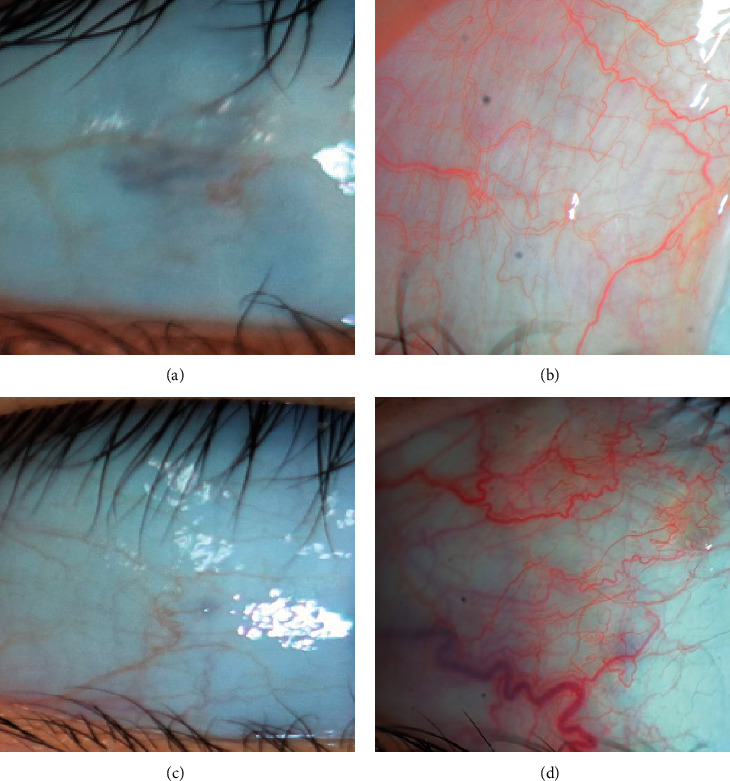
Anterior segment photographs of conjunctival injection before and after strabismus surgery in the brimonidine group ((a), (b)) and control group ((c), (d)). Conjunctival injection increased after strabismus surgery in both groups, while the amount of increase was less in the brimonidine group. Based on the preoperative conjunctival injection score, (b) scored 6.4 and (d) scored 33.3.

Conjunctival injection was measured by an objective method using software that automatically scores the region of interest from the image of the bulbar conjunctiva [16–18]. A masked observer measured each anterior segment photograph of the conjunctiva using the contrast-limited adaptive histogram equalization algorithm, and the results were converted into numeric values ranging from 0 to 100 (Figure 1). The nasal and temporal quadrants of the conjunctiva were analyzed separately. Extensive subconjunctival hemorrhage was not included in the region of interest. Postoperative conjunctival injection scores were assessed at 4 weeks after surgery and were calibrated as the amount of increase in numeric values compared with preoperative values.

### 2.4. Statistical Analysis

Statistical analysis was performed using SPSS version 22.0 for Windows (SPSS, Inc., Chicago). Independent t-test and chi-square test were used to compare the groups. Linear regression was used to analyze the relationship between conjunctival injection scores and age, cycloplegic refraction, angle of deviation, and surgical dosage. Statistical significance was set at *P* < 0.05. All continuous variables were reported as mean ± standard deviation (range, min, and max) values.

## 3. Results

A total of 63 consecutive patients (36 boys and 27 girls) who underwent RR surgery were included in the study. The mean age was 9.1 ± 1.0 years (range, 7.6–11.8 years). A comparison of clinical characteristics and surgical details between the two groups showed no statistically significant differences (Table 1). No cases of postoperative infection or abnormal bleeding occurred. No known adverse effects of brimonidine were reported, including dry mouth, fatigue/drowsiness, headache, sedation, hypotension, or bradycardia [19, 20].

### 3.1. Conjunctival Injection

**Figure 2:**
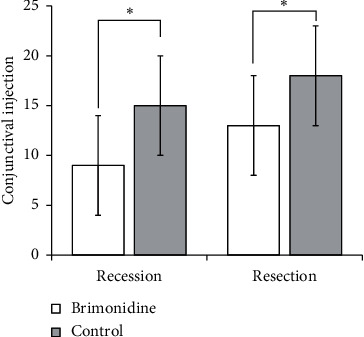
Comparison of conjunctival injection between the brimonidine group and control group. The mean score of injection at the temporal bulbar conjunctiva overlaying an area of the previous lateral rectus recession was significantly lower in the brimonidine group, 9.1 ± 9.9 in the brimonidine group and 15.9 ± 9.8 in the control group (*p*=0.008). At the nasal bulbar conjunctiva overlaying an area of the previous medial rectus resection, the mean score of conjunctival injection was significantly lower in the brimonidine group, 13.0 ± 11.1 in the brimonidine group and 18.6 ± 10.5 in the control group (*p*=0.046).

Figure 2 compares the differences in conjunctival injection scores between the two groups. The mean scores of postoperative conjunctival injection after rectus muscle recession and resection were significantly lower in the brimonidine group than the control group at 4 weeks after surgery (*P* = 0.008  and  0.046, respectively). In linear regression analysis, no linear correlation was observed between the surgical dosage, age, axial length, or the degree of conjunctival injection.

### 3.2. Intraocular Pressure

The mean intraocular pressure at 4 weeks after surgery was 11.7 ± 3.3 (range, 7–20) mmHg in the brimonidine group and 12.4 ± 3.0 (range, 8–19) mmHg in the control group (*P* = 0.418).

## 4. Discussion

In this study, we evaluated the safety and efficacy of treatment with 0.15% brimonidine tartrate ophthalmic solution for reducing conjunctival injection after strabismus surgery in children. These data demonstrate that the postoperative administration of topical brimonidine 0.15% can significantly reduce conjunctival injection after strabismus surgery.

Conjunctival injection is an important cosmetic problem after strabismus surgery. Escardo-Paton and Harrad [4] reported that the median duration of conjunctival injection following surgery was approximately 10 weeks. In addition, a few patients had persistent postoperative conjunctival injection beyond 24 weeks. Although postoperative conjunctival injection does not cause severe ophthalmological problems, it may be a cosmetic concern and cause psychosocial stress that affects social activities over several weeks or months, as would be required for the injection to subside [21].

Currently, the most widely used ophthalmic vasoconstrictors, such as phenylephrine, are *α*1- or mixed *α*1/*α*2-adrenergic receptor agonists. However, *α*1-adrenergic agonists are associated with cardiovascular adverse effects, and rebound conjunctival injection may occur when discontinued, which restricts their long-term use. [22–24] Conversely, since brimonidine affects vasoconstriction through the *α*2-adrenergic receptor, the possibility of cardiovascular events or rebound conjunctival injection is relatively low. Therefore, brimonidine can be safely used to reduce conjunctival injection after strabismus surgery. However, the overall safety profile of brimonidine in children under 2 years of age remains uncertain. [12] In our study, brimonidine was not prescribed for patients under 2 years of age.

Several studies have reported the efficacy of brimonidine in reducing bleeding-related problems in strabismus surgery [13–15]. Two previous studies prophylactically administered topical brimonidine or phenylephrine in patients with strabismus before surgery [13, 15]. Those studies reported that both drugs can reduce intraoperative bleeding and postoperative subconjunctival hemorrhage compared with sodium hyaluronate when assessed by a subjective scoring system [13, 15]. Dahlmann-Noor et al. [14]. analyzed video images up to 20 minutes after instilling brimonidine or apraclonidine at the beginning of adjustable strabismus surgery in 10 adults. They quantified the surface area of the anterior ocular blood vessels on video images with ImageJ (open source, public domain software) and reported that brimonidine reduced the surface area of blood vessels by 69.2% and this persisted for 20 minutes. In our study, we evaluated the efficacy of postoperative topical brimonidine 0.15% instillation for a prolonged period of 4 weeks in children using the contrast-limited adaptive histogram equalization (CLAHE) algorithm that automatically extracts vasculature and scores the degree of vascularity in numeric values ranging from 0 to 100, as performed in the previous studies [16–18]. We chose the CLAHE algorithm to determine postoperative conjunctival injection because it is theoretically most suitable for the quantification of vascularization, as shown in the previous studies [16–18, 25–27]. Our study is significant in that it has objectively proved the effect of brimonidine on conjunctival injection after strabismus surgery.

Topical brimonidine prescription has other benefits besides reducing conjunctival injection. Topical corticosteroids are generally used to control postoperative inflammation after strabismus surgery. However, there are concerns regarding IOP elevation associated with the use of steroids [17, 18, 28–31]. In particular, there have been several reports of increases in IOP by up to 82% due to topical steroids after strabismus surgery in children [30, 31]. Even fluorometholone, which causes lesser IOP elevation than other steroids, significantly induced IOP elevation after strabismus surgery in children, and 23% of patients reported an increase of ≥10 mm Hg compared with the baseline IOP [17]. The next-generation corticosteroid, loteprednol, can also cause IOP elevation after strabismus surgery, especially in young children ≤8 years of age [18]. Therefore, postoperative brimonidine can be a prophylactic measure in patients with steroid-induced IOP elevation.

Our study objectively confirmed that topical brimonidine can be used to control conjunctival injection after strabismus surgery in children aged 7 years or older. Furthermore, we found that brimonidine did not cause serious systemic complications in children. Brimonidine can also alleviate IOP elevation that may occur with topical steroid application after strabismus surgery.

There are some limitations that must be considered. First, our study was a nonrandomized retrospective study; therefore, there may be a selection bias. However, to exclude this bias, we included all patients who underwent RR surgery between September 2019 and April 2020. Second, since conjunctival injection was assessed at 4 weeks after using brimonidine, the long-term effect after stopping the drug remains to be elucidated.

In conclusion, administration of topical brimonidine 0.15% after strabismus surgery is efficacious and safe in reducing postoperative conjunctival injection. However, further research will be needed to determine the long-term effect of topical brimonidine on postoperative conjunctival injection after discontinuation of the drug.

## Figures and Tables

**Table 1:** Baseline characteristics of patients receiving topical brimonidine 0.15% and the control group.

Characteristics	Brimonidine (*n* = 32)	Control (*n* = 31)	Total (*n* = 63)	*P* value
Sex (M : F)	18 : 14	18 : 13	36 : 27	0.884^*∗*^
Laterality (R : L)	15 : 17	17 : 14	32 : 31	0.527^*∗*^
Mean age (years)	9.2 ± 1.1 (7.7–11.8)	9.0 ± 1.0 (7.6–11.6)	9.1 ± 1.0 (7.6–11.8)	0.549^†^
Cycloplegic refraction (D)	−1.30 ± 1.61 (−5.25–+2.25)	−1.34 ± 1.82 (−6.50–+1.25)	−1.32 ± 1.70 (−6.50–+2.25)	0.916^†^
*Angle of deviation (PD)*				
Distance	23.2 ± 7.4 (12−45)	22.8 ± 7.2 (12–45)	23.0 ± 7.2 (12–45)	0.824^†^
Near	29.9 ± 8.3 (15−45)	27.0 ± 8.6 (12–40)	28.5 ± 8.5 (12–45)	0.177^†^
*Surgical dosage (mm)*				
Medial rectus muscle	6.1 ± 0.9 (4.0–7.0)	5.7 ± 1.2 (3.5–7.0)	5.9 ± 1.0 (3.5–7.0)	0.168^†^
Lateral rectus muscle	5.6 ± 1.2 (4.0–8.5)	5.4 ± 1.2 (3.5–8.5)	5.5 ± 1.2 (3.5–8.5)	0.605^†^

*M* = male; *F* = female; *R* = right; *L* = left; *D* = diopters; PD = prism diopters.^*∗*^*P* value by Pearson's *χ*2 test. ^†^*P* value by independent t-test.

## Data Availability

The Institutional Review Board of Seoul National University Bundang Hospital/Ethics Committee has placed ethical restrictions to protect patient identities. However, the data are available to anyone who is interested without restriction. The minimal dataset will be available upon request. For data requests, please contact the SNUBH IRB office at 82-31-787-8804, 98614@snubh.org.
